# Study on deformation characteristics and crack extension law of cement stabilized phosphogypsum materials under dry and wet cycles

**DOI:** 10.1371/journal.pone.0327307

**Published:** 2025-07-24

**Authors:** Liangchun Xu, Kaisheng Chen, Yuang Chen, Hao Jian

**Affiliations:** 1 School of Civil Engineering, Guizhou University, Guiyang, Guizhou Province, China; 2 School of Civil Engineering, Southwest Jiaotong University, Sichuan Province, China; Shandong University of Technology, CHINA

## Abstract

The resource utilization of phosphogypsum has been a worldwide problem. Previous research was limited to mixing phosphogypsum with other materials (lime, gravel, red clay) to play an auxiliary role rather than being the main part, the utilization rate is low. This study aims to assess the deformation and crack behavior of cement-stabilized phosphogypsum under varying environmental conditions, and provide theoretical basis for the application of cement-stabilized phosphogypsum materials in road engineering. The test results showed that, firstly, the absolute expansion and absolute shrinkage of cement-stabilized phosphogypsum materials increased with the increase of compaction, cement dosage and the number of wet and dry cycles, and decreased with the increase of initial water content and the dosage of phosphogypsum; Secondly, the fracture rate of cement-stabilized phosphogypsum materials increased with cement dosage and the number of wet and dry cycles; and decreased with compaction, initial moisture content, and phosphogypsum dosage; Thirdly, the relationship between the absolute expansion or shrinkage of cement-stabilized phosphogypsum materials and the compaction degree and the fracture rate is not a simple linear relationship, but is affected by a combination of factors, showing nonlinear characteristics, which can be fitted by the nonlinear binary quadratic equation f(x,y) =ax^2^+bx+cy^2^+dy+e. The compaction degree and the fracture rate are the key factors influencing the material’s volume change, while the absolute expansion or shrinkage is a quantitative indicator of the volume change of the material under specific environmental conditions. Fitting by a nonlinear quadratic equation can effectively capture the complex nonlinear relationship between the variables and provide more accurate fitting results, and by analyzing the extreme points of this equation, the optimal degree of compaction can be determined to maximize the material properties.

## 2 Introduction

In China, most of the arable land lacks phosphorus, which is essential for crops and is one of the important factors affecting crop yields [1]. The annual demand for phosphorus fertilizer is about 8.5 million tons, and in general, the most important technology for producing phosphorus fertilizer is the wet process [2], Phosphogypsum is a solid waste discharged during the production of phosphorus fertilizer, and the treatment method is mostly open piles, while phosphogypsum contains CaSO₄·2H₂O also contains a small amount of phosphorus, fluorine, organic matter, oxides, and a small amount of heavy metals and radioactive substances and other impurities [3,4], which has a greater impact on the environment, which in turn jeopardizes human health. Usually every production of 1t of phosphate fertilizer need to discharge about 5t of phosphogypsum, China’s phosphogypsum production is large, although in recent years the utilization of phosphogypsum increased year by year, but the stock is still huge. Moreover, phosphogypsum reservoirs usually have high potential energy, which will not only cause serious ecological problems but also seriously threaten the life and property safety of the neighboring residents in the event of mudslides caused by dam failure [5]. The global annual phosphogypsum utilization rate is only 4.3% ~ 4.6%, and its stockpile has now exceeded 7 × 10^8^ t, and is still growing at a rate of about 8 × 10^7^ t per year. The massive accumulation of phosphogypsum not only encroaches on valuable land resources, but also causes serious pollution to the atmosphere, soil and water environment due to long-term weathering and rain erosion [6,7]. With the increasingly strict environmental regulations, phosphorus chemical enterprises are facing great environmental pressure, and there is an urgent need to find an effective way to deal with phosphogypsum.Applying phosphogypsum to road projects can effectively reduce the cost of highway construction. The use of this material reduces the dependence on traditional construction materials, thus saving money. And it can realize the resource utilization of phosphogypsum, reduce the negative impact of its accumulation on the environment, and largely solve the problem of low utilization of phosphogypsum, this utilization is in line with the concept of sustainable development, helps to promote the development of the circular economy, and has important academic research significance and practical application value [8].

The utilization of phosphogypsum can significantly reduce the pollution of industrial waste on the environment and promote the recycling of resources. The dry shrinkage coefficient of cement stabilized phosphogypsum material is low, about 50% of cement stabilized crushed stone mix, and it has better anti-cracking performance, which can effectively reduce the cracks of highway grass-roots level. Industrial waste phosphogypsum directly used in roadbed pavement filler can not meet the strength, deformation and other requirements, and its expansion and contraction characteristics are directly related to the stability, durability and driving safety of the pavement [9,10]. Phosphogypsum can be used as an efficient building material, reducing reliance on natural gypsum while reducing the environmental risks associated with phosphogypsum stockpiles. Ahmad et al [11] found the effect of addition of fly ash and fiber to foam concrete on its modulus of elasticity. Fly ash reduced the modulus of elasticity by 20%, while polypropylene fibers increased the modulus of elasticity at different densities. By adding phosphogypsum, the thermal conductivity of building insulation materials was significantly reduced, while the mechanical properties and durability of the materials were improved [12]. During the actual use of roads, the change of water content in the roadbed fill may lead to its contraction and expansion, especially in the dry season, the soil may contract due to water loss and form cracks. The generation of cracks will bring a series of hazards, such as causing deformation of the road surface and reduction of bearing capacity, etc. These hazards may affect the structural stability and service life of the road, therefore, many scholars have used different methods to improve the roadbed soil and reduce the generation of cracks [13,14]. Ahmad et al [15] found that permeable concrete was prepared by replacing coarse aggregate with waste glass particles and lightweight pumice aggregate and found that the lightweight aggregate improves the mechanical properties and permeability, but has lower abrasion resistance than waste glass particles. A study by Abdullah et al [16] tested the flexural strength of concrete-filled steel tubes (CFST) of different thicknesses and shapes and found that 2.0 mm thick CFSTs were stronger, rectangular CFSTs had a 92% increase in strength, and circular beams were more resistant to deflection, highlighting the importance of thicker plates and optimized design. Liu Zeyu [17] found that the absolute expansion and absolute shrinkage of phosphogypsum stabilized soil increased with the increase of compaction and cement dosage, and decreased with the increase of initial moisture content. Ji,Z. et al [18] revealed the strength decay mechanism, established a strength regression model, and explored the suitability as a pavement base (sub-base) material. Shallaw Abdulla et al [19] found that mineral admixtures can reduce carbon emissions from cement production, enhance concrete properties for sustainable construction and provide an important resource for the industry. Liu Zhihao [20] found that phosphogypsum can not only effectively improve the shrinkage properties and cracking resistance of cemented phosphogypsum-stabilized crushed stone, but also improve the splitting strength, compressive resilient modulus, and maximum dry shrinkage strain of stabilized crushed stone. Li Qiang [21] found that cement phosphogypsum can effectively improve the fissure extension characteristics of red clay. Analyzed from the perspective of fracture rate, cement phosphogypsum can inhibit the development of red clay fissures, and the inhibition effect is enhanced with the increase of phosphogypsum content, and the fracture rate is minimized when a ratio of phosphogypsum: red clay of 1:1. Stabilization of roadbed soil with rubber and plastic wastes increases its strength. The CBR was up to 50% at 10% plastic content and 28% at 5% rubber content, and this stabilization method reduces construction costs and pavement layer thickness [22]. From the above, it can be seen that the waste modification improves the concrete performance and the strength of the roadbed, and the phosphogypsum plays a certain inhibition effect on the expansion and contraction of the soil.

Liu Chun et al [23] developed a pore (particle) and fissure image recognition and analysis system (PCAS), which obtains binary images through operations such as multi-color segmentation and deblurring; implements three-dimensional sorting coefficients of the particle system calculated from the two-dimensional particle area; and uses probabilistic entropy and fractal dimensionality to characterize the directionality of the particles and pore space and the change of shape complexity, respectively, and so on. In other words, various geometric and statistical parameters of particles and pores can be obtained through simple operations. Scholars such as Liu Qinghe, Hao Jiaxin, and Han Hao calculated the cracks and pores of geotechnical bodies with the help of PCAS [24–26], and the method has been widely used for quantitative calculation of cracks and pores.

Based on the above studies, it can be seen that most of the research on the use of phosphogypsum as a road construction material is limited to the use of phosphogypsum mixed with other materials (lime, gravel, red clay, etc.), which only plays a supplementary role rather than being used as the main part, and mostly focused on the phosphogypsum roadbed pavement material strength and other mechanical properties of the study, the phosphogypsum deformation properties, crack expansion law of the study is rarely heard of, phosphogypsum utilization rate is low, a large number of piling up the problem can not be well solved. Therefore, in order to better solve these problems, this paper utilizes cement as stabilizer and highly doped phosphogypsum as stabilized object to carry out expansion and contraction and fissure expansion experiments, and considering the influence of climatic factors, different numbers of dry and wet cycles are added during the experimental process, so as to explore the absolute expansion rate under different numbers of dry and wet cycles, different degrees of compaction, different initial water contents, and different dosages of cement through the fitting of experimental data, shrinkage and fissure rate. And through the nonlinear fitting of the relationship between the absolute expansion or shrinkage of the mixture and the compaction degree and the fracture rate, the deformation behavior of cement stabilized phosphogypsum materials is more accurately described and predicted to reduce the generation of fissures and increase the service life of the road, which provides a scientific basis for the application of cement stabilized phosphogypsum materials in the road project and improves the reliability and durability of the project.

## 3 Nature of raw materials

The phosphogypsum used in this test was taken from urnfu phosphorus mine in Guizhou province, which is white-gray in color after drying, and we have made a detailed study on the basic physical indexes and chemical composition of phosphogypsum as shown in Tables 1 and 2, and the results of heavy metal and radioactivity testing are shown in Table 3, and the heavy metal content of phosphogypsum complies with the requirements of the national standards of GB 5085.3−2007 “Hazardous Waste Identification Standards for Toxicity Identification by Leaching” after the test, and the radioactivity index also meets the requirements of national standards (GB 6566−2010).

**Table 1:** Basic Parameters of Phosphogypsum.

Specific surface area /(m^2^‧kg^-1^)	Loss on ignition/%	Moisture content/%	Alkali content/%	Density /(g‧cm^-3^)	fineness /%
103	19.32	5.36	1.33	2.86	43.9

**Table 2:** Chemical Composition of Phosphogypsum.

Ingredient	SO_3_	CaO	SiO_2_	P_2_O_5_	Na_2_O	Al_2_O_3_	Other
Mass fraction/%	48.237	40.649	5.854	2.130	0.631	0.173	2.326

**Table 3:** Test Results of Heavy Metals and Radioactivity in Phosphogypsum.

Test items		Standard limits	Result	Conclusion
Heavy metal	Cu/(mg‧L^-1^)	≤100	0.142	Qualified
	Zn/(mg‧L^-1^)	≤100	0.047	Qualified
	Cd/(mg‧L^-1^)	≤1	0	Qualified
	Pb/(mg‧L^-1^)	≤5	0	Qualified
	Cr/(mg‧L^-1^)	≤15	0	Qualified
	As/(mg‧L^-1^)	≤5	0.0342	Qualified
	Hg/(mg‧L^-1^)	≤0.1	0.0004	Qualified
Radioactivity	Ra-226/(Bq‧kg^-1^)	—	55.86	—
	TH-232/(Bq‧kg^-1^)	—	42.28	—
	K-40/(Bq‧kg^-1^)	—	51.52	—
	I_Ra_	≤1.0	0.3	Qualified
	I_γ_	≤1.0	0.3	Qualified

The cement used in this experiment is Southwest brand P.C32.5R silicate cement, which is gray in color and dry, and its basic parameters are shown in Table 4.

**Table 4:** Basic parameters of cement.

Loss on ignition/%	Chloride ion /%	Sulfur trioxide/%	Alkali/%	Initial setting time/min	Final setting time/min
1.76	0.020	2.83	3.78	304	324
3d $f_{cf}$/MPa	28d $f_{cf}$/MPa	3d $f_{cu}$/MPa	28d $f_{cu}$/MPa	Stability	
5.2	6.8	25.7	44.3	Qualified	

## 4 Design of mix ratios

### 4.1 Mixing ratio design

Through reviewing the previous research literature [27–29] and combining with the purpose of this study, in order to make phosphogypsum can be used in large dosage in road projects, and effectively solve the problem of low utilization of phosphogypsum, so the dosage of phosphogypsum is controlled at about 90%, and because the higher the dosage of cement is, the higher the cost of the material will be, so the dosage range of cement for cement-stabilized phosphogypsum base layer is controlled at 6%−12%, and the corresponding dosage of phosphogypsum is 94%−88%. The dosage range of cement stabilized phosphogypsum base material is 6%−12%, and the corresponding phosphogypsum dosage is 94%−88%. When using cement stabilized soil for subgrade for construction, if the plasticity index is higher than 12, the Technical Rules for Highway Pavement Construction (JTG/T F20-2015) [30] stipulates that the mixing ratio of cement should be limited to 6% to 14%, so the proposed cement mixing amount for this test is 5%, 7% and 9%. By reviewing the previous research literature [31,32] and combining the specification “Highway pavement grass-roots construction technical rules” (JTG/T F20-2015) [30] on the compaction of cement stabilized class materials, the compaction degree of the third and fourth grade highway is not less than 90%, and the compaction degree of the first grade highway is not less than 96%, and considering different parts of different grades of highway roadbase, the set of the three compaction degrees are 94%, 96%, 98%. The optimum water content and maximum dry density of the specimen obtained through the compaction test are shown in Table 5.According to the experience of previous scholars, the soil structure tends to be stabilized after 4 ~ 6 times of wet and dry cycles [33], and this test increases the number of wet and dry cycles on the basis of this test, so the proposed number of wet and dry cycles in this test is 7 times.

**Table 5:** Optimal Moisture Content and Maximum Dry Density of Mixture under Different Cement Content.

cement dosage/%	Optimum moisture content/%	Maximum dry density/g·cm^-3^
0	13.79	1.431
5	14.22	1.470
7	14.56	1.515
9	14.00	1.486

### 4.2 Sample preparation

**Fig 1:**
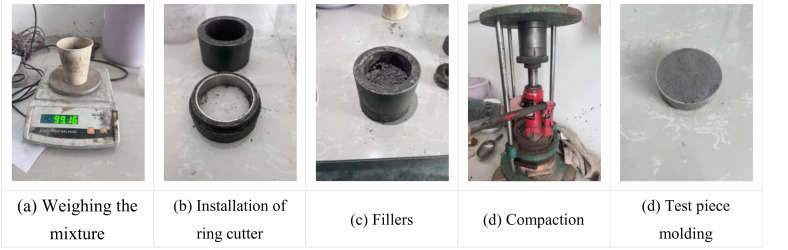
Sample preparation process diagram.

Phosphogypsum was dried before making samples, and after drying, it was pounded with a hammer and passed through a 2 mm geotechnical standard sieve, and its moisture content after drying was determined by alcohol combustion method. Weigh a certain mass of phosphogypsum, according to the design ratio mixing materials, and according to the optimal moisture content should be added to the calculation of the amount of water, reserved 10% of the amount of water after the remaining water evenly mixed into the material. After mixing uniformly, use cling film to seal for 24h to ensure the water content in the mixture is uniform. Then the predetermined mass of cement and reserved water were added to the mixture and mixed again, after mixing well and passing through the 2 mm geotechnical standard sieve again, the corresponding mass of the mixture was weighed, and the ring cutter, ring cutter specimen maker and jack were used for ring cutter specimen preparation. The detailed sample preparation process is shown in Fig 1.

## 5 Pilot programs

### 5.1 Expansion and contraction test

All the tests involved in this study were conducted in the laboratory of the School of Civil Engineering, Guizhou University. All test procedures were approved by the laboratory management of the School of Civil Engineering, Guizhou University, prior to the commencement of the study. The specimens for this experiment were prepared as samples according to Table 6.The mixture was put into the ring cutter, and after precise mixing, the ring cutter specimen was made, and the specimen was placed in an environment where the temperature was kept at 20 ± 2°C and the humidity was not less than 95% for maintenance for 7-day age. The ring blade specimen was securely embedded in the collar, the specimen was weighed in its entirety and the data recorded, then it was placed in a soil expansion meter equipped with a percentage gauge, making sure that the measuring probe of the gauge was centered on the soil sample, and the initial readings were subsequently taken and recorded. After the specimen is properly installed, perform the wet and dry cycle test repeatedly and record the results of each cycle in detail. During the wet and dry cycling process of the specimen, the humidification stage is characterized by the absolute expansion rate ($\delta_{ae}$); the drying process is characterized by the absolute shrinkage rate ($\delta_{as}$). The specific equations were calculated according to equations (1) and (2):

**Table 6:** Specimen Preparation.

groups	cement dosage/%	compaction/%	moisture content/%	Number of wet and dry cycles
A	5	94、96、98	14.22(Optimum moisture content)	1 ~ 7
	7		14.56(Optimum moisture content)	
	9		14.00(Optimum moisture content)	
B	7	94、96、98	9.56	1 ~ 7
			14.56(Optimum moisture content)	
			19.56	


$$\begin{array}{c} \delta_{ae}=\frac{h_{ei}\;-\;h_{0}}{h_{0}}\times 100\%\;\; \end{array}$$ (1)

Note: $\delta_{ae}=$ absolute expansion rate; $h_{ei}=$ Height of the specimen after the ith expansion stabilization; $h_{0}=$ Initial height of the specimen.


$$\begin{array}{c} \delta_{as}=\frac{h_{si}\;-\;h_{0}}{h_{0}}\times 100\%\;\;\; \end{array}$$ (2)

Note: $\delta_{as}=$ absolute shrinkage rate; $h_{si}=$ The height of the specimen after stabilization of the ith contraction; $h_{0}=$ Initial height of the specimen.

### 5.2 Fissure expansion test

**Fig 2:**
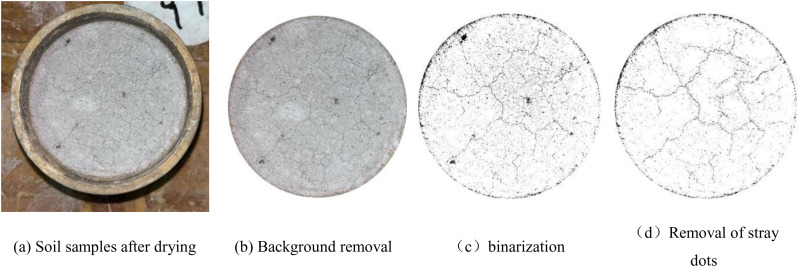
Image processing process.

All the tests involved in this study were conducted in the laboratory of the School of Civil Engineering, Guizhou University. All test procedures were approved by the laboratory management of the School of Civil Engineering, Guizhou University, prior to the commencement of the study. The specimens for this experiment were prepared as samples according to Table 6. The specimens were prepared according to subsection 2.2, and the fracture extension test was performed according to Liu Zeyu and Chen Kaisheng [34,35]. After each wet and dry cycle, the specimens were photographed using a camera, which was required to keep the same distance between the camera and the specimen in each photo. PCAS (Particle and Fissure Image Recognition and Analysis System) was used to recognize and process the fissure photographs taken [23], and the processing is shown in Fig 2.

First, the background of the photographs was removed, and the circular part of the specimen surface was retained. After removing the background, the image was binarized, and the grayscale value of the image was adjusted by setting a threshold so that the cracks were clearly visible, and the image was divided into a black area (the crack area) and a white area (the soil block area). The image processed by binarization will show non-crack clutter in the white area, so it is necessary to remove these clutter by image processing techniques. The fracture ratio is used as a quantitative index to compare the development of crack in specimens under different conditions. The fissure rate is the percentage of the fissure area of the specimen surface to the total surface area of the specimen, and the black pixel area in the image by binarization is the fissure area and the white pixel area is the total area of the specimen, which is calculated as shown in equation (3).


$$\begin{array}{c} \delta =\sum_{i=0}^{n}\frac{A_{i}}{A}\;\;\; \end{array}$$ (3)

Note: δ= Fracture ratio; i = Fissure numbering; Ai = Article i Crack area; A = Total area of specimen.

### 5.3 Dry and wet cycle test program

The group of 2020 presided over the completion of the National Natural Science Foundation of China project “dry and wet cycle of red clay slope damage mechanism and protection technology research”, a site slope water content monitoring for a period of one year, the monitoring results of the slope water content of 25% to 50% amplitude change. Chen Nan, Wu Lijian [36] and others carried out tests on the water content of slopes in a total of 33 locations in six sections of Guizhou Yukai Highway and Kaiyang Highway, and the water content of slopes ranged from 16% to 45%. Therefore, according to the actual data of on-site monitoring by the group and the research results of literature [31], considering the extreme climate environment, the dry and wet cycle amplitude of the water content rate is determined to be between 10% and 50%, and the optimal water content rate of phosphogypsum tested in this paper is 13.79%, so it is proposed that the dry and wet cycle amplitude is set at 45%. It is proposed that the humidification to the target moisture content is set at 50% and the drying to the target moisture content is set at 5%. By carrying out the pre-test, after adding water and standing for 24 hours, the moisture content of each layer of the specimen (top, middle and bottom) was stabilized within the range of 50% ± 1%, indicating that the moisture distribution within the specimen was relatively uniform. As the water is immersed from the upper part of the specimen, the internal water content of the specimen has a certain difference in the longitudinal direction, which is manifested in the higher water content of the upper layer, while the middle and lower layers of the water content is relatively low, but are close to the upper limit of the water content. Therefore, in the formal test stage, each group of specimens can use the same water injection calculation method, and rest for 24 hours to ensure the uniformity of the test conditions.

**Fig 3:**
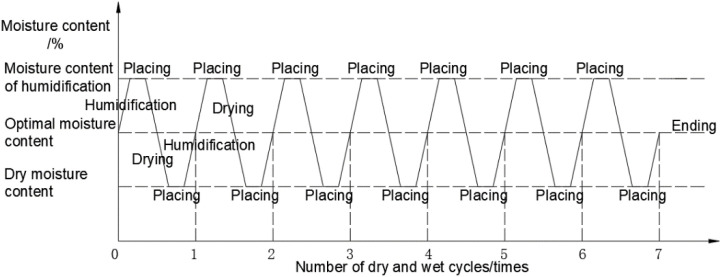
Schematic diagram of the wet/dry cycle process.

**Fig 4:**
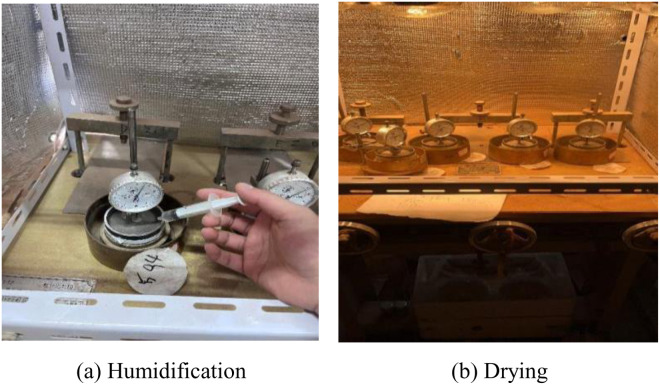
Dry wet cycle process.

The specimens were cured and then subjected to wet-dry cyclic tests in the manner of Fig 3. According to the research of Long Anfa, Li Zhen and Xie Huihui [37–39], the strength attenuation of the soil body after experiencing first wet and then dry is greater than that of first dry and then wet, so this paper adopts the method of first wet and then dry to carry out the wet and dry cycle test. The humidification process was carried out by syringe injection; the drying process was carried out by turning on the bath heater and weighing the mass of the specimen at 1h intervals until the target moisture content was reached. (1) humidification stage: weigh the calculated mass of water, use a syringe to inject slowly, humidification time for the specimen to freely absorb water for 24h, so that the water is sufficiently homogeneous to read the micrometer readings; (2) Drying stage: The specimen is dried to the target moisture content by turning on the bath heater, and the moisture content of the specimen is monitored by weighing the specimen by removing the specimen as a whole every hour during the drying process. The process of drying and wetting cycle is shown in Fig 4.

## 6 Analysis of test results

### 6.1 Absolute expansion rate of the mix

#### (1) Relationship between absolute expansion rate and degree of compaction.

**Fig 5:**
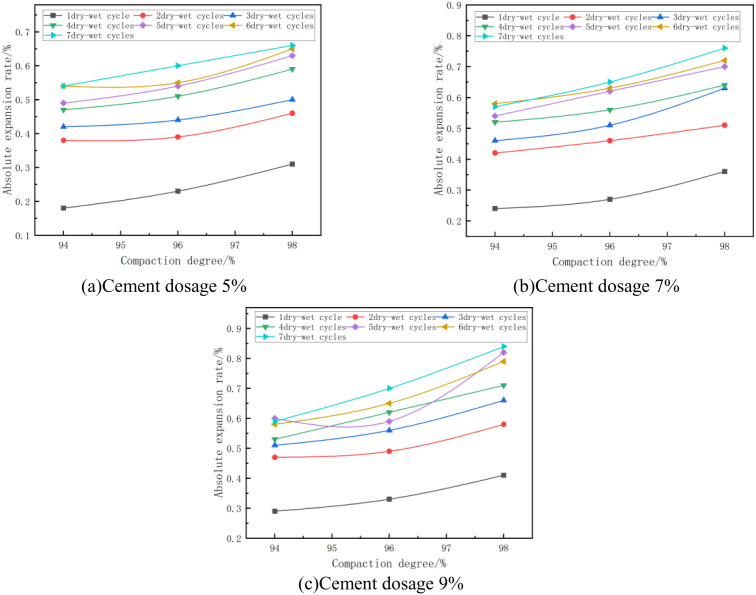
Absolute expansion rate versus compaction.

The absolute expansion of the mixes at optimum moisture content and at different degrees of compaction is shown in Fig 5. From Fig 5, it can be seen that the absolute expansion rate increases with the increase of compaction degree. Taking Fig 5(a) with cement dosage of 5% and 4 wet and dry cycles as an example, when the compaction degree of the specimen increases from 94% to 96%, its absolute expansion rate changes from 0.47% to 0.51%, with an increase of 6%, and when the compaction degree of the specimen increases from 96% to 98%, its absolute expansion rate changes from 0.51% to 0.59%, with an increase of 16%. This is due to the fact that when preparing the specimen, as compaction rises, the specimen’s interior gets denser, the smaller the spacing between its soil particles, the greater the inter-particle interaction force, the easier it is for expansion and deformation to occur during the expansion experiment, and the greater the absolute expansion rate is after the expansion is stabilized [40,41]. Meanwhile, according to Ji Xiaoping et al [42], it was found that phosphogypsum contains hydrophilic mineral Na_2_SiF_6_, and the larger the compaction degree of the specimen, the more soil particles it contains in the same volume, and the hydrophilic mineral content contained in the mixed material increases, which enhances the expansion potential inside the specimen, and makes the specimen with a large degree of compaction swell more when it is absorbing water to increase humidity, and on the contrary, when the specimen’s degree of compaction is small, the soil particles inside it and and the hydrophilic material is less. On the contrary, when the compaction degree of the specimen is small, its internal soil particles and and hydrophilic materials are less, and the specimen swells less when absorbing water and humidifying, which further proves that the absolute expansion rate of the specimen increases with the increase of compaction degree.

#### (2) Relationship between absolute expansion rate and initial moisture content.

**Fig 6:**
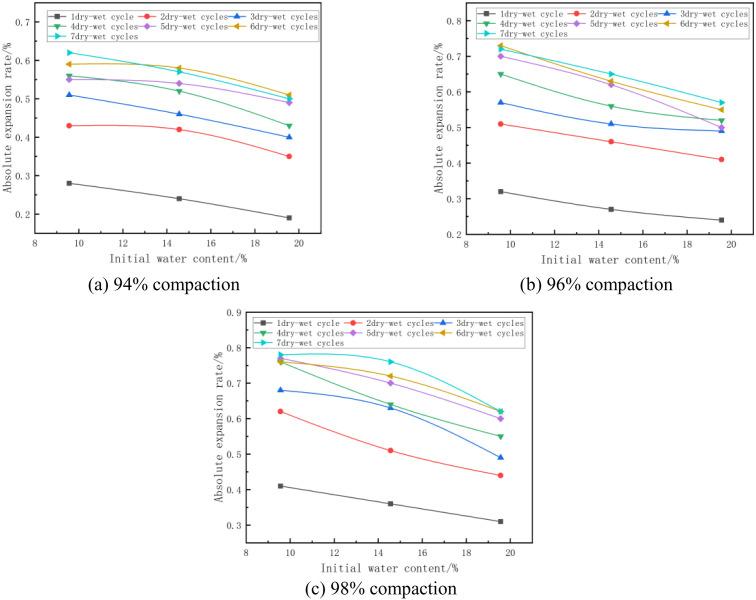
Absolute expansion rate versus initial water content.

The absolute expansion of the mixes at a cement dose of 7% and at different initial water contents is shown in Fig 6. From Fig 6, it can be seen that the absolute expansion of the specimen decreases with the increase of initial water content. Taking Fig 6(c) with 7% cement dosage, 98% compaction, and 6 wet and dry cycles as an example, when the initial water content of the specimen increased from 14.56% (optimum water content) to 19.56% (optimum water content + 5%), its absolute expansion rate changed from 0.72% to 0.62%, which was reduced by 0.1%. This is due to the same mixture of water absorption are the same, the initial water content of the lower specimen water absorption after saturation of the combined water film thickness is greater, while the water will be mixed particles “chiseled open”, so that the specimen expansion deformation is greater, so in order to ensure that the compaction of the mixture in the case of raising the initial water content of the mixture can effectively reduce the expansion and deformation of the mixture [43].

#### (3) The relationship between absolute expansion rate and cement dosage.

**Fig 7:**
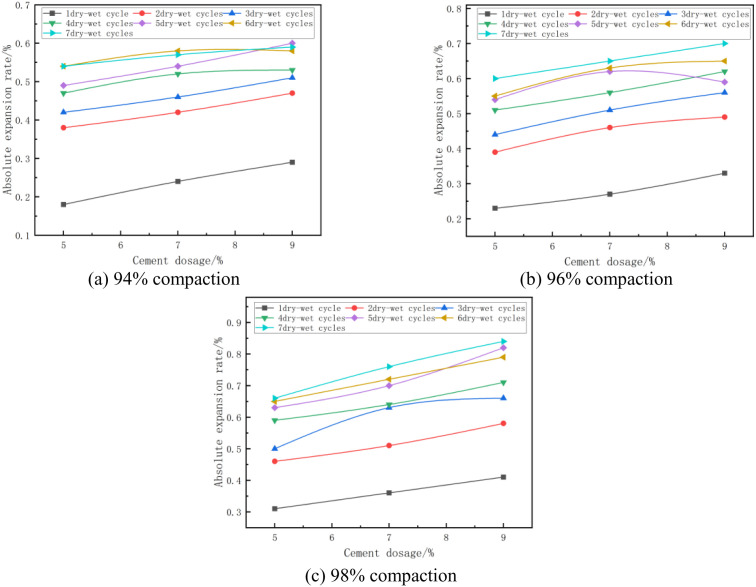
Absolute expansion rate versus cement dosage.

The absolute expansion of the mixes at optimum moisture content and different cement doses is shown in Fig 7. From Fig 7, it can be seen that the absolute expansion of the specimen increases with the increase of cement dose. Taking Fig 7(b) with 96% compaction and 7 wet and dry cycles as an example, the absolute expansion rate of the specimen is 0.60% when the cement dose of the specimen is 5%, and 0.65% when the cement dose of the specimen is 7%, which is an increase of 8%, which is due to the fact that the hydration reaction of the cement produces AFt, hydrated calcium silicate (C-S-H) gels, and hydrated calcium aluminum (C-A-H), During cement hydration, AFt first covers the surface of cement particles and then grows outward in a radial pattern until the crystal ends come into contact with other particles, which allows expansion forces to be generated between the crystal layers [44].

#### (4) Relationship between absolute expansion rate and number of wet and dry cycles.

**Fig 8:**
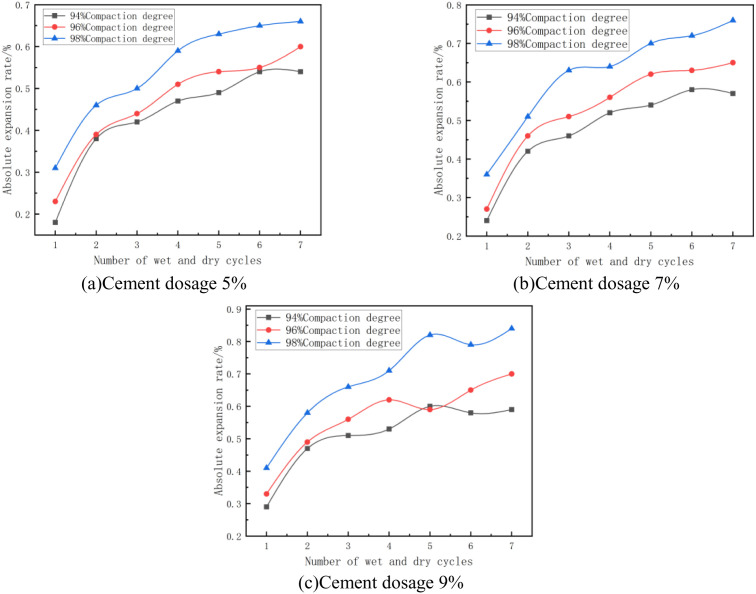
Absolute expansion rate versus number of wet and dry cycles.

The absolute expansion rate of the mix at the optimum moisture content and different number of wet and dry cycles is shown in Fig 8. As can be seen from Fig 8, the absolute expansion rate of the specimen with the increase in the number of wet and dry cycles showed a trend of first increasing and then stabilizing, the growth trend is manifested as: rapid growth – slow growth – stabilization of three stages. After 1 ~ 2 dry and wet cycles, the absolute expansion rate of the specimen grows rapidly, 3 ~ 5 dry and wet cycles grow slowly, 6 ~ 7 dry and wet cycles tend to stabilize, and the height of the specimen remains basically unchanged. Taking Fig 7(a) with 5% cement dosage and 98% compaction as an example, the absolute expansion rate of the specimen changed from 0.31% to 0.46% after 1 ~ 2 dry and wet cycles, which increased by 0.15%, this is due to the fact that in the initial stage of dry and wet cycles, the structure inside the specimen did not reach a stable state, and the soil sample’s closed pore space was gradually transformed to the connecting pore space, and the porosity increased rapidly, because the pore space increase led to the increase of the inter-particle Because the increase of pore space leads to the increase of inter-particle voids and thus provides more space for expansion, cracks are generated inside the soil samples [45,46], leading to the increase of absolute expansion rate; After 4–5 wet and dry cycles, its absolute expansion rate changed from 0.52% to 0.63%, with an increase of 21%; after 6–7 wet and dry cycles, the absolute expansion rate changed from 0.65% to 0.66%, with an increase of 2%. This is because as the number of wet and dry cycles increases, the porosity, particle arrangement, crack size and number, and the products of chemical reactions within the specimen change less, so the absolute expansion rate of the mix tends to stabilize.

### 6.2 Absolute shrinkage rate of the mix

#### (1) Relationship between absolute shrinkage rate and degree of compaction.

**Fig 9:**
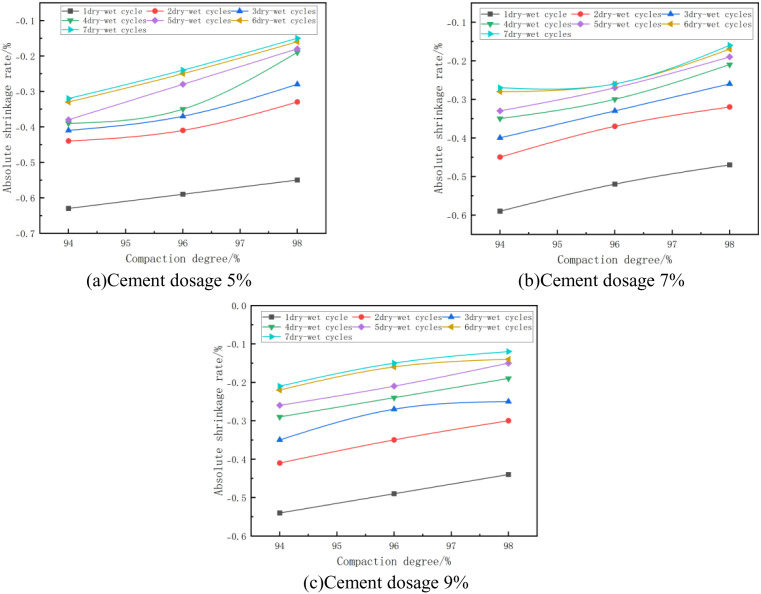
Absolute shrinkage rate versus compaction.

The absolute shrinkage of the mixes at optimum moisture content and at different degrees of compaction is shown in Fig 9. From Fig 9, it can be seen that the absolute shrinkage increases with the increase of compaction degree. As an example, in Fig 9(a) with 5% cement dosage and 2 wet and dry cycles, the absolute shrinkage changes from −0.44% to −0.41%, an increase of 7%, when the compaction of the specimen increases from 94% to 96%, and from −0.41% to −0.33%, an increase of 24%, when the compaction of the specimen increases from 96% to 98%. This is due to the fact that with the increase of compaction, the contact between the particles becomes closer and the hydration products generated by the cement hydration reaction are distributed more uniformly inside the material, and the friction and binding forces between the particles are significantly increased, and this increased binding force creates a higher stress concentration inside the material. Especially during the drying process, the evaporation of water leads to volumetric shrinkage of the hydration products, which causes greater absolute shrinkage [47,48].

#### (2) Relationship between absolute shrinkage rate and initial water content.

**Fig 10:**
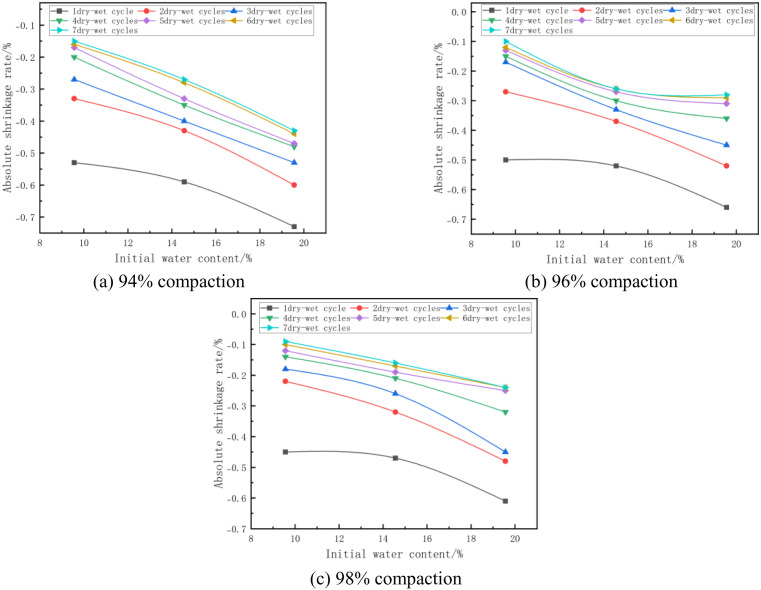
Absolute shrinkage rate versus initial water content.

The absolute shrinkage of the mixes at 7% cement dose and different initial water content is shown in Fig 10. From Fig 10, it can be seen that the absolute shrinkage decreases with the increase of initial water content. Taking Fig 10(b) with 96% compaction and 3 wet and dry cycles as an example, when the initial moisture content of the specimen increases from 9.56% (optimal moisture content −5%) to 14.56% (optimal moisture content), its absolute shrinkage changes from −0.17% to −0.33%, which is reduced by 0.15%, and when the initial moisture content of the specimen increases from 14.56% (optimal moisture content) to 19.56% (optimum moisture content +5%), its absolute shrinkage changed from −0.33% to −0.45%, which decreased by 0.12%, this is due to the fact that when the moisture content increases, the pores inside the material are filled with water, reducing the air content in the pores and thus reducing the capillary action of the pores. Capillary action is one of the main causes of drying shrinkage and therefore an increase in moisture content leads to a decrease in the absolute shrinkage of the mix [49].

#### (3) Relationship between absolute shrinkage rate and cement dosage.

**Fig 11:**
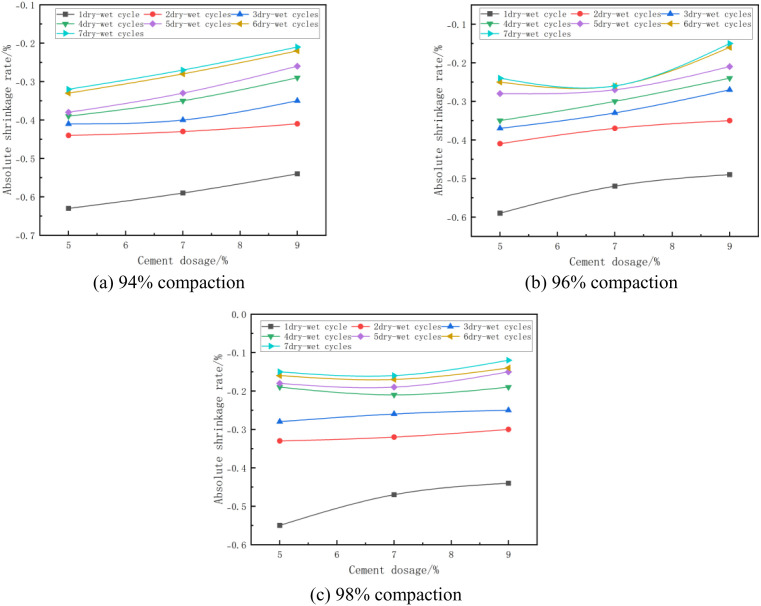
Absolute shrinkage rate versus cement dosage.

The absolute shrinkage of the mixes at optimum moisture content and different cement doses is shown in Fig 11. From Fig 11, it can be seen that the absolute shrinkage of the specimen increases with the increase of cement dose. As an example, in Fig 11(a) with 94% compaction and 5 wet and dry cycles, the absolute shrinkage of the specimen is −0.38% when the cement dosage of the specimen is 5% and −0.33% when the cement dosage of the specimen is 7%, which is an increase of 15%. This is due to the fact that as the cement dosing increases, the hydration products generated during cement hydration (e.g., C-S-H gel and AFt) also increase, and these hydration products fill the pores within the material, making the microstructure of the material denser. While this densification increases the strength of the material, it also increases the concentration of stresses within the material. When water evaporates, these stresses lead to more significant shrinkage deformation of the material [49], which results in an increase in the absolute shrinkage of the mix.

#### (4) Relationship between absolute shrinkage rate and number of wet and dry cycles.

**Fig 12:**
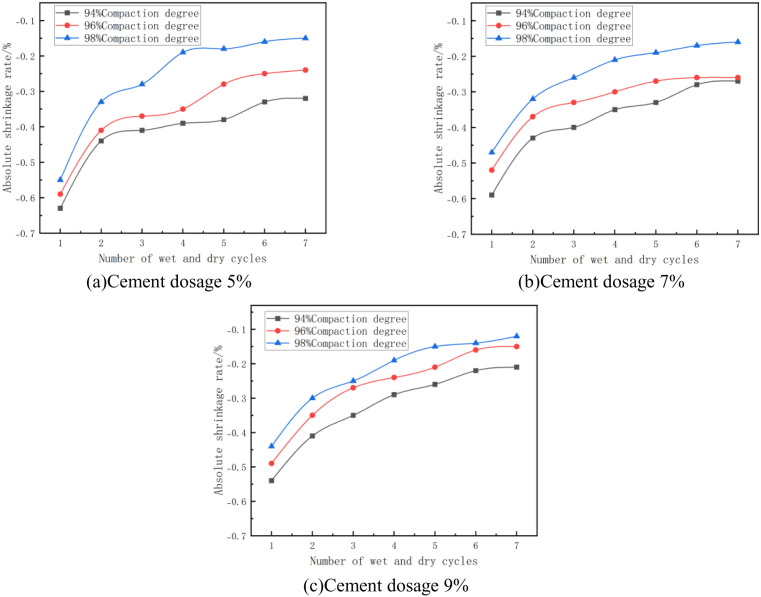
Absolute shrinkage rate versus number of wet and dry cycles.

The absolute shrinkage of the mixes at optimum moisture content and different number of wet and dry cycles is shown in Fig 12. From Fig 12, it can be seen that the absolute shrinkage of the specimen increases with the increase of the number of wet and dry cycles, and all of them show a trend of rapid increase first, and then gradually leveled off. Taking Fig 12(b) with 96% compaction as an example, the absolute shrinkage of the specimen increased from −0.52% to −0.37% after 1 ~ 2 dry and wet cycles, with an increase of 29%; after 3 ~ 5 dry and wet cycles, the absolute shrinkage increased from −0.33% to −0.27%, with an increase of 18%, with a decrease in the growth rate; and after 6 ~ 7 dry and wet cycles, the absolute shrinkage was all – 0.26%, with no increase, at which time the absolute expansion rate remained unchanged. This indicates that with the increase of the number of dry and wet cycles, the shrinkage and deformation of the specimen is getting smaller and stabilized.

### 6.3 Fissure expansion test

#### (1) Crack development.

**Fig 13:**
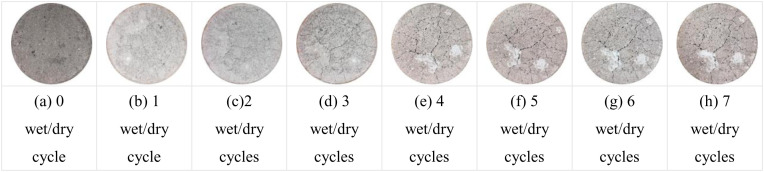
7% cement dosage 96% compaction degree Crack development in 7 wet and dry cycles.

**Fig 14:**
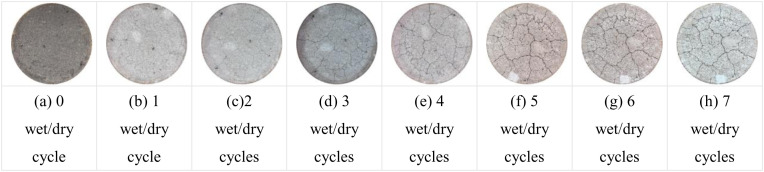
7% cement dosage 98% compaction degree Crack development in 7 wet and dry cycles.

Qualitative comparative analysis of the pattern of crack generation and expansion of specimens with 7% cement dosage at optimum water content and 96% and 98% compaction under different number of wet and dry cycles is shown in Figs 13 and 14. As can be seen from Fig 13, the specimen started to show fine cracks on the surface after 1 wet and dry cycle; with the increase of the number of wet and dry cycles, the cracks started to expand and their number increased; The cracks in the original basis of widening and deepening extension, 4 times after the wet and dry cycle can be clearly seen on the surface of the specimen gap, and the surface of a small part of the white area, this area is the specimen surface rupture shedding formation. The specific performance of the specimen after 1 dry and wet cycle, the surface of the specimen began to crack; 2 ~ 4 dry and wet cycle, the specimen shows that the cracks began to expand, the number of cracks increased, the cracks began to gradually become wider, the visual effect is more obvious; 5 ~ 7 dry and wet cycle crack development has been basically stabilized, the width of the cracks basically remain unchanged. As can be seen from Fig 13 and Fig 14, the crack development of the two groups of specimens is more significant after wet and dry cycling, and these cracks are gradually expanded and deepened during wet and dry cycling, forming an obvious network of cracks. The distribution of cracks is not uniform, and the cracks are more intensive in some areas, showing the local damage of the material under the action of dry and wet cycling.

#### (2) Relationship between fracture ratio and degree of compaction.

**Fig 15:**
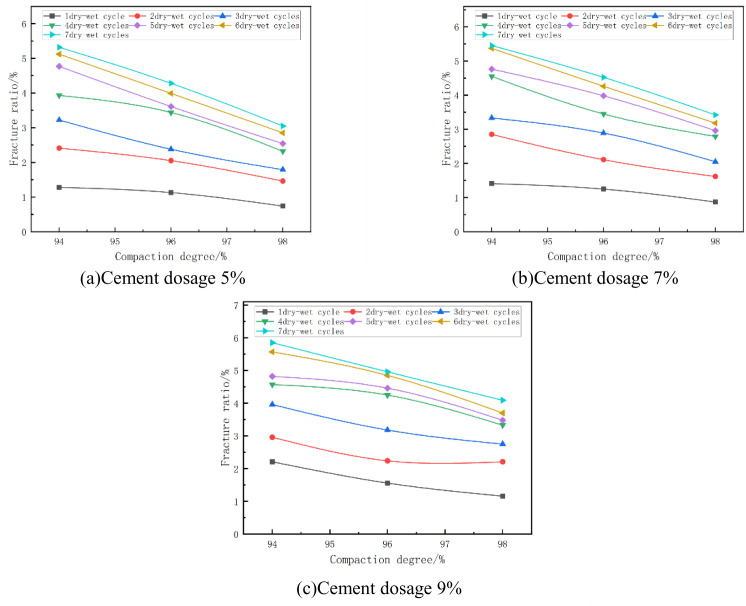
Fracture ratio versus compaction degree.

The fracture rate of the mixes at optimum moisture content and at different degrees of compaction is shown in Fig 15. From Fig 15 it can be seen that the fracture rate decreases with the increase of compaction. As an example, in Fig 15(b) with cement dosage of 7% and 3 wet and dry cycles, when the compaction of the specimen increased from 94% to 96%, its fracture rate changed from 3.33% to 2.89%, which was reduced by 13%, and when the compaction of the specimen increased from 96% to 98%, its fracture rate changed from 2.89% to 2.05%, which was reduced by 29%. The fracture rate and depth of fracture development of cement-stabilized phosphogypsum materials decreased significantly with increasing compaction, and the overall development of fractures weakened. However, due to the centralization of the crack distribution, the cracks may be more obvious at the macroscopic level. This change reflects the combined effect of compaction on the microstructure and mechanical behavior of the material [50].

#### (3) Relationship between fracture ratio and initial water content.

**Fig 16:**
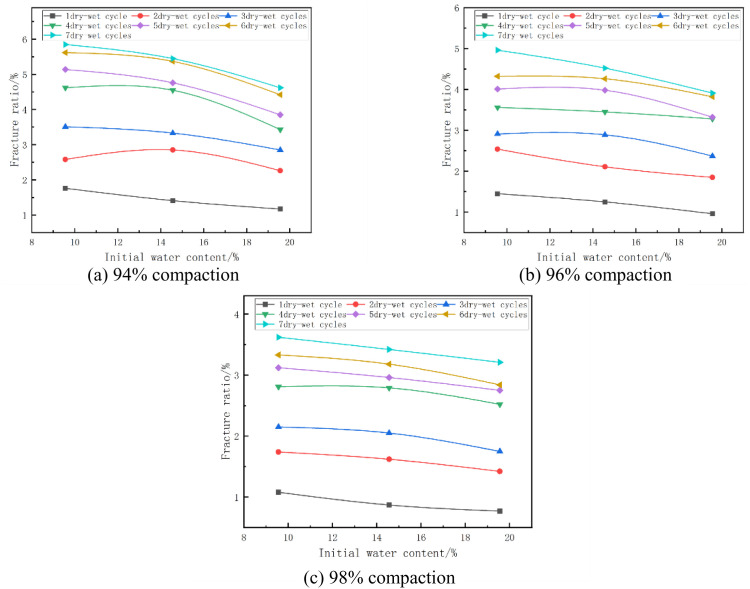
Fracture ratio versus initial water content.

The absolute expansion of the mixes at a cement dose of 7% and at different initial water contents is shown in Fig 16. From Fig 16, it can be seen that the fracture rate of the specimen decreases with the increase of initial water content. Taking Fig 16(c) with 98% compaction and 5 wet and dry cycles as an example, when the initial water content of the specimen increased from 9.56% (optimal water content −5%) to 14.56% (optimal water content), its fracture rate changed from 3.12% to 2.96%, which decreased by 5%, and when the initial water content of the specimen increased from 14.56% (optimal water content) to 19.56% (optimal water content + 5%), its fracture rate changed from 2.96% to 2.75%, which decreased by 7%. This is due to the fact that as the moisture content increases, the moisture distribution within the material becomes more uniform, the hydration reaction of the cement becomes more adequate, and the fracture rate decreases significantly.

#### (4) Relationship between fracture ratio and cement dosage.

**Fig 17:**
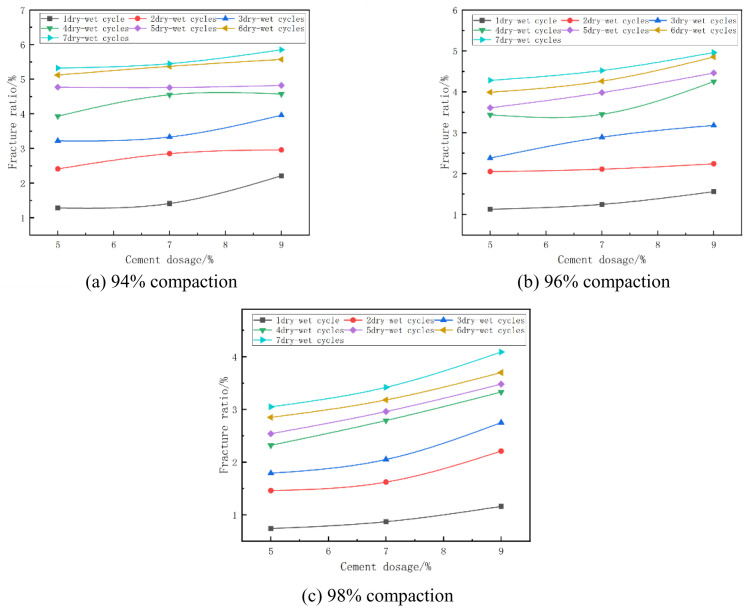
Fracture ratio versus cement dosage.

The fracture ratio of the mixes at optimum moisture content and at different cement doses is shown in Fig 17. From Fig 17, it can be seen that the fracture rate of the specimen increases with the increase of cement dose. For example, in Fig 17(b) with 96% compaction and 5 wet and dry cycles, the fracture rate of the specimen increased from 3.61% to 3.98% when the cement dose of the specimen was increased from 5% to 7%, which is an increase of 10%, and the fracture rate of the specimen increased from 4.46% to 4.46% when the cement dose of the specimen was increased from 7% to 9%, which is an increase of 11%. This is due to the fact that the amount of hydration products increases when the cement dose is increased. These hydration products not only fill the pores, but are also able to wrap around the surface of the particles and enhance the inter-particle bonding. This filling effect reduces the space for the development of microscopic defects and cracks within the material, thus reducing the fracture rate of the specimens [51].

#### (5) Relationship between fracture ratio and number of wet and dry cycles.

**Fig 18:**
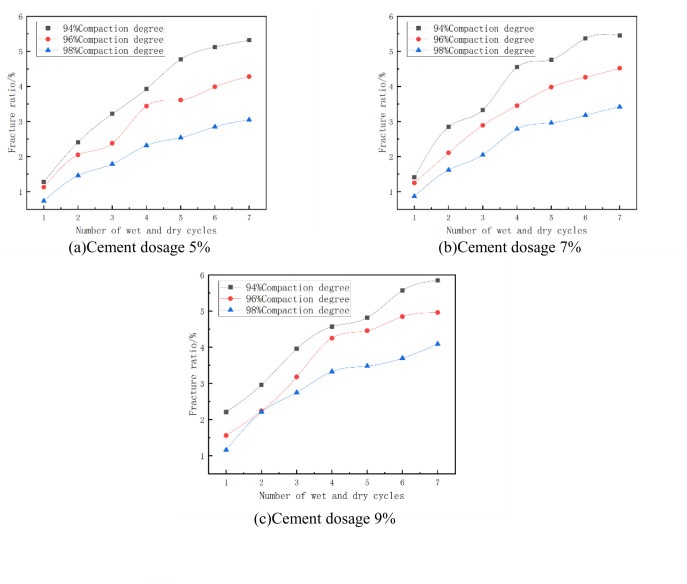
Fracture ratio versus number of wet and dry cycles.

The absolute expansion of the mixes at optimum moisture content and different number of wet and dry cycles is shown in Fig 18. From Fig 18, it can be seen that with the increase of the number of dry and wet cycles, the cleavage of cement phosphogypsum stabilized material is the first to increase gradually and then tend to stabilize. In 1 ~ 3 times of wet and dry cycles, the specimen fissure rate increases rapidly; after 4 ~ 5 times of wet and dry cycles, the increase is slow; 6 ~ 7 times of wet and dry cycles tend to stabilize. Under the action of dry and wet cycles, the fracture rate of cement phosphogypsum materials increases with the increase of the number of cycles, which is because with the formation of the fracture network, the stress concentration point decreases, the driving force of the new fracture in the subsequent cycles is weakened, and the rate of fracture expansion decreases, and ultimately tends to the dynamic equilibrium. This phenomenon indicates that the microcracks in the initial stage gradually expand and interconnect with the increase in the number of cycles to form macroscopic cracks, leading to a rapid increase in the fracture rate. With the formation of the fracture network, the stress distribution inside the material is gradually homogenized, the driving force for fracture expansion is weakened, and the increase in fracture rate is gradually stabilized [52,53].

### 6.4 Nonlinear fitting of absolute expansion rate (shrinkage rate) of mixes to compaction and fracture ratio

According to the rule of change of absolute expansion (shrinkage) rate of cement phosphogypsum stabilized material under dry and wet cycles derived from the previous section and from the study of crack expansion law of cement phosphogypsum stabilized material, it can be seen that there exists a certain correlation between the two, so on this basis to further explore the mathematical relationship between deformation indexes and their influencing factors, by integrating the deformation indexes and crack indexes, to construct a function-fitting model. In the Technical Rules for Highway Pavement Subgrade Construction (JTG/T F20-2015) [30], specific requirements are set for the compaction degree of pavement subgrade materials for different grades of highways, so the compaction degree can be considered as the dependent variable. Through our many attempts, we found that choosing the quadratic form of fitting has a high degree of fit, and the correlation coefficients are very high in the results of absolute expansion and contraction fitting in Tables 7 and 8, indicating a better fit.

**Table 7:** Absolute expansion rate fitting results.

Cement dosage Coefficient			
	5%	7%	9%
a	0.0099	0.00863	0.00998
b	−1.84133	−1.59051	−1.84646
c	−0.02111	−0.02112	−0.02273
d	0.22394	0.23036	0.25191
e	85.56607	73.1972	85.28187
R^2^	0.97363	0.97772	0.94861

**Table 8:** Absolute shrinkage rate fitting results.

Cement dosage Coefficient			
	5%	7%	9%
f	0.01143	0.00364	−0.00156
g	−2.12825	−0.64979	0.35111
h	−0.02876	0.00111	−0.00987
i	0.26156	0.03985	0.16036
j	98.14199	28.38005	−20.0691
R^2^	0.95429	0.92301	0.97462

**Fig 19:**
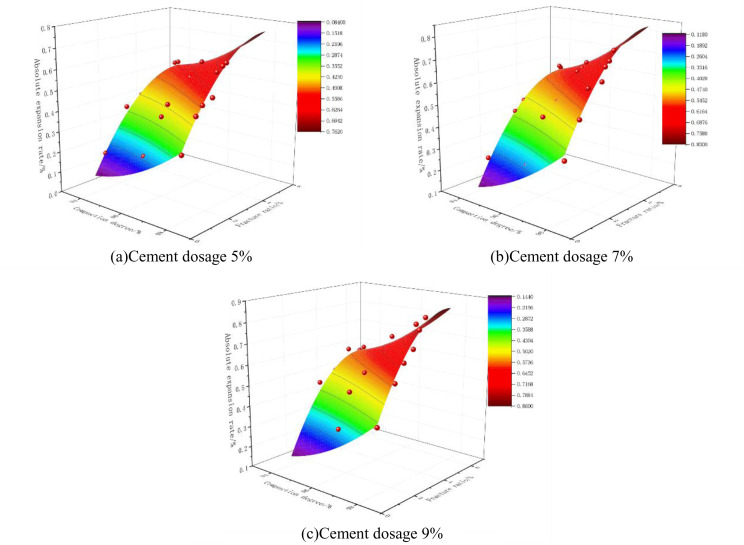
Absolute expansion rate fitting results.


The fitting equation of the absolute expansion of the specimen as a function of its compaction and fracture ratio is shown in Eq. (4), and the fitting results are shown in Table 7 and Fig 19.

$$\begin{array}{c} M=ax^{2}+bx+cy^{2}+dy+e\;\; \end{array}$$ (4)

Note:M = Absolute expansion rate (%);x=Compaction (%);y= fracture ratio(%);a,b,c,d,e= polynomial coefficients.

The fitting equation of the absolute shrinkage rate of the specimen as a function of its compaction and fracture ratio is shown in equation (5):


$$\begin{array}{c} N=fx^{2}+gx+hy^{2}+iy+j\;\; \end{array}$$ (5)

Note:N = Absolute shrinkage rate (%);x=Compaction (%);y= fracture ratio(%);f,g,h,i,j= polynomial coefficients.

**Fig 20:**
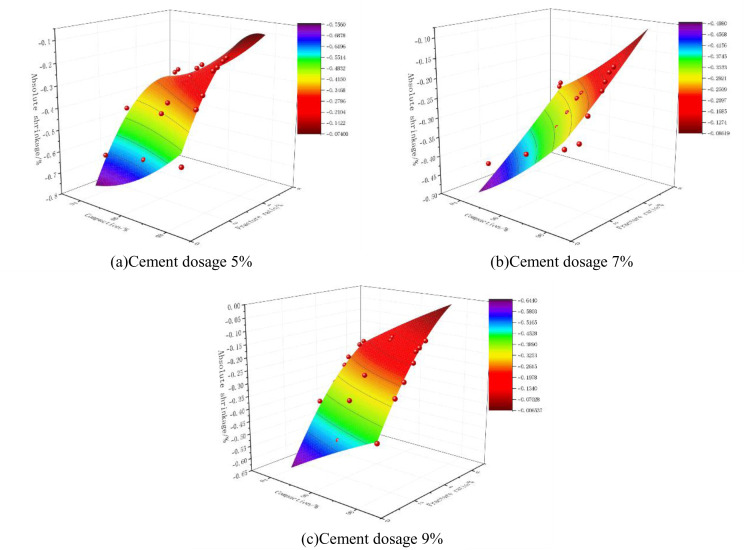
Absolute shrinkage rate fitting results.

The results of the fit are shown in Table 8 and Fig 20.

R^2^ in Tables 7 and 8 is a goodness-of-fit indicator, and the closer its value is to 1, the better the fit, and in general, its value is not less than 0.9, which means a good fit. As can be seen from Tables 7 and 8, the values of R^2^ in this fit are all greater than 0.9, indicating that this nonlinear binary quadratic equation is able to fit the relationship between compaction, fracture ratio, and absolute expansion and contraction or shrinkage better.

## 7 Conclusion

In this paper, the deformation characteristics and fissure expansion law of cement-stabilized phosphogypsum under dry and wet cycling were investigated through the experimental tests of expansion and contraction as well as the image processing system PCAS, and the following conclusions were obtained:

(1) The absolute expansion and absolute shrinkage of cement-stabilized phosphogypsum materials increase with the increase of compaction, cement dosage and the number of wet and dry cycles, and decrease with the increase of initial moisture content and the dosage of phosphogypsum;
(2) The fracture ratio of cement-stabilized phosphogypsum material increases with the increase of cement dosage and the number of wet and dry cycles; and decreases with the increase of compaction, initial moisture content, and phosphogypsum dosage. By incorporating phosphogypsum into the mixture, the stability of the internal structure of the mixture can be increased and the extension of the cracks of the mixture can be inhibited, which is a great help to the research on the use of phosphogypsum for roadbed filler.
(3) The relationship between the absolute expansion or shrinkage of cement-stabilized phosphogypsum materials and compaction and fracture ratio can be fitted by a nonlinear quadratic equation.

This paper is closely related to the major needs of national highway construction, and actively responds to the call of Guizhou Provincial People’s Government and Guiyang Municipal People’s Government on the comprehensive utilization of phosphogypsum resources, and studies the deformation characteristics and fissure expansion law of cement-stabilized phosphogypsum materials under dry-wet cycle with high doping phosphogypsum as the premise and considering the complex climatic characteristics of dry-wet cycle, but there is still a great limitation:

(1) In this paper, the deformation characteristics of cement-stabilized phosphogypsum materials under dry and wet cycles are carried out under no-load conditions, while in the actual road pavement is usually subject to the influence of vehicle extrusion, so the subsequent research can be considered to carry out the relevant tests under load conditions. Modern testing technology has been able to accurately simulate the effects of vehicle loads on road base materials. Dynamic Mechanical Analyzers (DMA) and tri-axial testing machines can simulate the effects of loads of different frequencies and amplitudes, and study the deformation and fatigue characteristics of materials under cyclic loading. Under the action of load, the strength characteristics of materials will change, accelerating the deformation of materials, and cyclic loading will lead to fatigue damage of materials [54]. Through the load test, the performance changes of the material in the construction process can be monitored in real time, and potential problems can be found and solved in time to improve the service life and reliability of the road.
(2) In this paper, the characteristics of cement phosphogypsum stabilized materials used in pavement base materials are mainly investigated through indoor experiments, and there is a lack of field experiments, so the next step can be to carry out research on the relevant contents of the actual project from the field experiments. Construction techniques for cement-stabilized phosphogypsum materials include processes such as mixing, paving and compaction of the materials, which can be effectively applied to field tests, and the required construction equipment (e.g., pavers, rollers) and monitoring equipment (e.g., nondestructive testing equipment, automated monitoring systems) are widely available on the market and can meet the needs of field tests. Test sections of different lengths and widths can be designed, with different material ratios and construction techniques, respectively, for comparative studies to provide optimization suggestions for actual construction [55]. Field tests can more accurately assess the deformation characteristics, durability and strength characteristics of materials in actual use. Through long-term monitoring, it can understand the change rule of material performance in actual use, develop a more scientific maintenance strategy, reduce the occurrence of road diseases, and reduce maintenance costs.

In summary, this research adopts a combination of experimental and numerical simulation methods to systematically study the deformation characteristics and crack extension law of cement-stabilized phosphogypsum materials under dry and wet cycling conditions, and the research results not only provide scientific basis and technical support for road engineering, but also provide important theoretical and practical guidance for sustainable construction, which largely solves the problems of low utilization of phosphogypsum and disordered stockpiling.

## Supporting information

S1 File Minimum data set. (DOCX)
