# Supplementary material for: Study on deformation characteristics and crack extension law of cement stabilized phosphogypsum materials under dry and wet cycles
Source: PLoS One. 2025 Jul 24;20(7):e0327307. doi: 10.1371/journal.pone.0327307 (PMC12289013; doi:10.1371/journal.pone.0327307)
Supplement: S1 File — (DOCX) [file pone.0327307.s001.docx]

**Minimum data set**

**S1 Table Basic Parameters of Phosphogypsum**

| Specific surface area  / (m^2^‧kg^-1^) | Loss on ignition /% | Moisture content /% | Alkali content /% | Density  /(g‧cm^-3^) | fineness  /% |
| --- | --- | --- | --- | --- | --- |
| 103 | 19.32 | 5.36 | 1.33 | 2.86 | 43.9 |

**S2 Table Chemical Composition of Phosphogypsum**

| Ingredient | SO_3_ | CaO | SiO_2_ | P_2_O_5_ | Na_2_O | Al_2_O_3_ | Other |
| --- | --- | --- | --- | --- | --- | --- | --- |
| Mass fraction/% | 48.237 | 40.649 | 5.854 | 2.130 | 0.631 | 0.173 | 2.326 |

**S3 Table Test Results of Heavy Metals and Radioactivity in Phosphogypsum**

| Test items | | Standard limits | Result | Conclusion |
| --- | --- | --- | --- | --- |
| Heavy metal | Cu/(mg‧L^-1^) | ≤100 | 0.142 | Qualified |
|  | Zn/(mg‧L^-1^) | ≤100 | 0.047 | Qualified |
|  | Cd/(mg‧L^-1^) | ≤1 | 0 | Qualified |
|  | Pb/(mg‧L^-1^) | ≤5 | 0 | Qualified |
|  | Cr/(mg‧L^-1^) | ≤15 | 0 | Qualified |
|  | As/(mg‧L^-1^) | ≤5 | 0.0342 | Qualified |
|  | Hg/(mg‧L^-1^) | ≤0.1 | 0.0004 | Qualified |
| Radioactivity | Ra-226/(Bq‧kg^-1^) | — | 55.86 | — |
|  | TH-232/(Bq‧kg^-1^) | — | 42.28 | — |
|  | K-40/(Bq‧kg^-1^) | — | 51.52 | — |
|  | I_Ra_ | ≤1.0 | 0.3 | Qualified |
|  | I_γ_ | ≤1.0 | 0.3 | Qualified |

**S4 Table Basic parameters of cement**

| Loss on ignition /% | Chloride ion  /% | Sulfur trioxide /% | Alkali /% | Initial setting time /min | Final setting time /min |
| --- | --- | --- | --- | --- | --- |
| 1.76 | 0.020 | 2.83 | 3.78 | 304 | 324 |
| 3d $f_{cf}$/MPa | 28d $f_{cf}$/MPa | 3d $f_{cu}$/MPa | 28d $f_{cu}$/MPa | Stability |  |
| 5.2 | 6.8 | 25.7 | 44.3 | Qualified |  |

**S5 Table Optimal Moisture Content and Maximum Dry Density of Mixture under Different Cement Content**

| cement dosage /% | Optimum moisture content /% | Maximum dry density /g·cm^-3^ |
| --- | --- | --- |
| 0 | 13.79 | 1.431 |
| 5 | 14.22 | 1.470 |
| 7 | 14.56 | 1.515 |
| 9 | 14.00 | 1.486 |

**S6 Table** **Cement dose 5%. Absolute expansion rate of the mix/%**

| Number of wet and dry cycles/N | 94% compaction | 96% compaction | 98% compaction |
| --- | --- | --- | --- |
| 1 dry wet cycle | 0.18 | 0.23 | 0.31 |
| 2 dry wet cycles | 0.38 | 0.39 | 0.46 |
| 3 dry wet cycles | 0.42 | 0.44 | 0.50 |
| 4 dry wet cycles | 0.47 | 0.51 | 0.52 |
| 5 dry wet cycles | 0.49 | 0.54 | 0.63 |
| 6 dry wet cycles | 0.54 | 0.55 | 0.65 |
| 7 dry wet cycles | 0.54 | 0.60 | 0.66 |

**S7 Table Cement dose 7%. Absolute expansion rate of the mix/%**

| Number of wet and dry cycles/N | 94% compaction | 96% compaction | 98% compaction |
| --- | --- | --- | --- |
| 1 dry wet cycle | 0.24 | 0.27 | 0.36 |
| 2 dry wet cycles | 0.42 | 0.46 | 0.51 |
| 3 dry wet cycles | 0.46 | 0.51 | 0.63 |
| 4 dry wet cycles | 0.52 | 0.56 | 0.64 |
| 5 dry wet cycles | 0.54 | 0.62 | 0.70 |
| 6 dry wet cycles | 0.58 | 0.63 | 0.72 |
| 7 dry wet cycles | 0.57 | 0.65 | 0.76 |

**S8 Table Cement dose 9%. Absolute expansion rate of the mix/%**

| Number of wet and dry cycles/N | 94% compaction | 96% compaction | 98% compaction |
| --- | --- | --- | --- |
| 1 dry wet cycle | 0.29 | 0.33 | 0.41 |
| 2 dry wet cycles | 0.47 | 0.49 | 0.58 |
| 3 dry wet cycles | 0.51 | 0.56 | 0.66 |
| 4 dry wet cycles | 0.53 | 0.62 | 0.71 |
| 5 dry wet cycles | 0.60 | 0.59 | 0.82 |
| 6 dry wet cycles | 0.58 | 0.65 | 0.79 |
| 7 dry wet cycles | 0.59 | 0.70 | 0.84 |

**S9 Table Compaction 94%. Absolute expansion rate of the mix/%**

| Number of wet and dry cycles/N | Initial moisture content 9.56% | Initial moisture content 14.56% | Initial moisture content 19.56% |
| --- | --- | --- | --- |
| 1 dry wet cycle | 0.28 | 0.24 | 0.19 |
| 2 dry wet cycles | 0.43 | 0.42 | 0.35 |
| 3 dry wet cycles | 0.51 | 0.46 | 0.40 |
| 4 dry wet cycles | 0.56 | 0.52 | 0.43 |
| 5 dry wet cycles | 0.55 | 0.54 | 0.49 |
| 6 dry wet cycles | 0.59 | 0.58 | 0.51 |
| 7 dry wet cycles | 0.62 | 0.57 | 0.50 |

**S10 Table Compaction 96%. Absolute expansion rate of the mix/%**

| Number of wet and dry cycles/N | Initial moisture content 9.56% | Initial moisture content 14.56% | Initial moisture content 19.56% |
| --- | --- | --- | --- |
| 1 dry wet cycle | 0.32 | 0.27 | 0.24 |
| 2 dry wet cycles | 0.51 | 0.46 | 0.41 |
| 3 dry wet cycles | 0.57 | 0.51 | 0.49 |
| 4 dry wet cycles | 0.65 | 0.56 | 0.52 |
| 5 dry wet cycles | 0.70 | 0.62 | 0.50 |
| 6 dry wet cycles | 0.73 | 0.63 | 0.55 |
| 7 dry wet cycles | 0.72 | 0.65 | 0.57 |

**S11 Table Compaction 98%. Absolute expansion rate of the mix/%**

| Number of wet and dry cycles/N | Initial moisture content 9.56% | Initial moisture content 14.56% | Initial moisture content 19.56% |
| --- | --- | --- | --- |
| 1 dry wet cycle | 0.41 | 0.36 | 0.31 |
| 2 dry wet cycles | 0.62 | 0.51 | 0.44 |
| 3 dry wet cycles | 0.68 | 0.63 | 0.49 |
| 4 dry wet cycles | 0.76 | 0.64 | 0.55 |
| 5 dry wet cycles | 0.77 | 0.70 | 0.60 |
| 6 dry wet cycles | 0.76 | 0.72 | 0.62 |
| 7 dry wet cycles | 0.78 | 0.76 | 0.62 |

**S12 Table Cement dose 5%. Absolute shrinkage rate of the mix/%**

| Number of wet and dry cycles/N | 94% compaction | 96% compaction | 98% compaction |
| --- | --- | --- | --- |
| 1 dry wet cycle | -0.63 | -0.59 | -0.55 |
| 2 dry wet cycles | -0.44 | -0.41 | -0.33 |
| 3 dry wet cycles | -0.41 | -0.37 | -0.28 |
| 4 dry wet cycles | -0.39 | -0.35 | -0.19 |
| 5 dry wet cycles | -0.38 | -0.28 | -0.18 |
| 6 dry wet cycles | -0.33 | -0.25 | -0.16 |
| 7 dry wet cycles | -0.32 | -0.24 | -0.15 |

**S13 Table Cement dose 7%. Absolute shrinkage rate of the mix/%**

| Number of wet and dry cycles/N | 94% compaction | 96% compaction | 98% compaction |
| --- | --- | --- | --- |
| 1 dry wet cycle | -0.59 | -0.52 | -0.47 |
| 2 dry wet cycles | -0.43 | -0.37 | -0.32 |
| 3 dry wet cycles | -0.40 | -0.33 | -0.26 |
| 4 dry wet cycles | -0.35 | -0.30 | -0.21 |
| 5 dry wet cycles | -0.33 | -0.27 | -0.19 |
| 6 dry wet cycles | -0.28 | -0.26 | -0.17 |
| 7 dry wet cycles | -0.27 | -0.26 | -0.16 |

**S14 Table Cement dose 9%. Absolute shrinkage rate of the mix/%**

| Number of wet and dry cycles/N | 94% compaction | 96% compaction | 98% compaction |
| --- | --- | --- | --- |
| 1 dry wet cycle | -0.54 | -0.49 | -0.44 |
| 2 dry wet cycles | -0.41 | -0.35 | -0.30 |
| 3 dry wet cycles | -0.35 | -0.27 | -0.25 |
| 4 dry wet cycles | -0.29 | -0.24 | -0.19 |
| 5 dry wet cycles | -0.26 | -0.21 | -0.15 |
| 6 dry wet cycles | -0.22 | -0.16 | -0.14 |
| 7 dry wet cycles | -0.21 | -0.15 | -0.12 |

**S15 Table Compaction 94%. Absolute shrinkage rate of the mix/%**

| Number of wet and dry cycles/N | Initial moisture content 9.56% | Initial moisture content 14.56% | Initial moisture content 19.56% |
| --- | --- | --- | --- |
| 1 dry wet cycle | -0.53 | -0.59 | -0.73 |
| 2 dry wet cycles | -0.33 | -0.43 | -0.60 |
| 3 dry wet cycles | -0.27 | -0.40 | -0.53 |
| 4 dry wet cycles | -0.20 | -0.35 | -0.48 |
| 5 dry wet cycles | -0.17 | -0.33 | -0.47 |
| 6 dry wet cycles | -0.16 | -0.28 | -0.44 |
| 7 dry wet cycles | -0.15 | -0.27 | -0.43 |

**S16 Table Compaction 96%. Absolute shrinkage rate of the mix/%**

| Number of wet and dry cycles/N | Initial moisture content 9.56% | Initial moisture content 14.56% | Initial moisture content 19.56% |
| --- | --- | --- | --- |
| 1 dry wet cycle | -0.50 | -0.52 | -0.66 |
| 2 dry wet cycles | -0.27 | -0.37 | -0.52 |
| 3 dry wet cycles | -0.17 | -0.33 | -0.45 |
| 4 dry wet cycles | -0.15 | -0.30 | -0.36 |
| 5 dry wet cycles | -0.13 | -0.27 | -0.31 |
| 6 dry wet cycles | -0.12 | -0.26 | -0.29 |
| 7 dry wet cycles | -0.10 | -0.26 | -0.28 |

**S17 Table Compaction 98%. Absolute shrinkage rate of the mix/%**

| Number of wet and dry cycles/N | Initial moisture content 9.56% | Initial moisture content 14.56% | Initial moisture content 19.56% |
| --- | --- | --- | --- |
| 1 dry wet cycle | -0.45 | -0.47 | -0.61 |
| 2 dry wet cycles | -0.22 | -0.32 | -0.48 |
| 3 dry wet cycles | -0.18 | -0.26 | -0.45 |
| 4 dry wet cycles | -0.14 | -0.21 | -0.32 |
| 5 dry wet cycles | -0.12 | -0.19 | -0.25 |
| 6 dry wet cycles | -0.10 | -0.17 | -0.24 |
| 7 dry wet cycles | -0.09 | -0.16 | -0.24 |

**S18 Table Cement dose 5%. Fracture ratio of the mix/%**

| Number of wet and dry cycles/N | 94% compaction | 96% compaction | 98% compaction |
| --- | --- | --- | --- |
| 1 dry wet cycle | 1.28 | 1.13 | 0.74 |
| 2 dry wet cycles | 2.41 | 2.05 | 1.46 |
| 3 dry wet cycles | 3.22 | 2.38 | 1.79 |
| 4 dry wet cycles | 3.93 | 3.44 | 2.32 |
| 5 dry wet cycles | 4.77 | 3.61 | 2.54 |
| 6 dry wet cycles | 5.12 | 3.99 | 2.85 |
| 7 dry wet cycles | 5.32 | 4.28 | 3.05 |

**S19 Table Cement dose 7%. Fracture ratio of the mix/%**

| Number of wet and dry cycles/N | 94% compaction | 96% compaction | 98% compaction |
| --- | --- | --- | --- |
| 1 dry wet cycle | 1.41 | 1.25 | 0.87 |
| 2 dry wet cycles | 2.85 | 2.11 | 1.62 |
| 3 dry wet cycles | 3.33 | 2.89 | 2.05 |
| 4 dry wet cycles | 4.55 | 3.45 | 2.79 |
| 5 dry wet cycles | 4.76 | 3.98 | 2.96 |
| 6 dry wet cycles | 5.37 | 4.26 | 3.18 |
| 7 dry wet cycles | 5.45 | 4.52 | 3.42 |

**S20 Table Cement dose 9%. Fracture ratio of the mix/%**

| Number of wet and dry cycles/N | 94% compaction | 96% compaction | 98% compaction |
| --- | --- | --- | --- |
| 1 dry wet cycle | 2.21 | 1.56 | 1.16 |
| 2 dry wet cycles | 2.96 | 2.24 | 2.21 |
| 3 dry wet cycles | 3.96 | 3.18 | 2.75 |
| 4 dry wet cycles | 4.57 | 4.25 | 3.33 |
| 5 dry wet cycles | 4.82 | 4.46 | 3.48 |
| 6 dry wet cycles | 5.57 | 4.85 | 3.70 |
| 7 dry wet cycles | 5.85 | 4.96 | 4.09 |

**S21 Table Compaction 94%. Fracture ratio of the mix/%**

| Number of wet and dry cycles/N | Initial moisture content 9.56% | Initial moisture content 14.56% | Initial moisture content 19.56% |
| --- | --- | --- | --- |
| 1 dry wet cycle | 1.76 | 1.41 | 1.17 |
| 2 dry wet cycles | 2.58 | 2.85 | 2.26 |
| 3 dry wet cycles | 3.51 | 3.33 | 2.85 |
| 4 dry wet cycles | 4.62 | 4.55 | 3.43 |
| 5 dry wet cycles | 5.14 | 4.76 | 3.85 |
| 6 dry wet cycles | 5.62 | 5.37 | 4.42 |
| 7 dry wet cycles | 5.85 | 5.45 | 4.62 |

**S22 Table Compaction 96%. Fracture ratio of the mix/%**

| Number of wet and dry cycles/N | Initial moisture content 9.56% | Initial moisture content 14.56% | Initial moisture content 19.56% |
| --- | --- | --- | --- |
| 1 dry wet cycle | 1.45 | 1.25 | 0.96 |
| 2 dry wet cycles | 2.54 | 2.11 | 1.85 |
| 3 dry wet cycles | 2.91 | 2.89 | 2.37 |
| 4 dry wet cycles | 3.56 | 3.45 | 3.28 |
| 5 dry wet cycles | 4.01 | 3.98 | 3.32 |
| 6 dry wet cycles | 4.32 | 4.26 | 3.82 |
| 7 dry wet cycles | 4.96 | 4.52 | 3.91 |

**S23 Table Compaction 98%. Fracture ratio of the mix/%**

| Number of wet and dry cycles/N | Initial moisture content 9.56% | Initial moisture content 14.56% | Initial moisture content 19.56% |
| --- | --- | --- | --- |
| 1 dry wet cycle | 1.08 | 0.87 | 0.77 |
| 2 dry wet cycles | 1.74 | 1.62 | 1.42 |
| 3 dry wet cycles | 2.15 | 2.05 | 1.75 |
| 4 dry wet cycles | 2.81 | 2.79 | 2.52 |
| 5 dry wet cycles | 3.12 | 2.96 | 2.75 |
| 6 dry wet cycles | 3.33 | 3.18 | 2.84 |
| 7 dry wet cycles | 3.62 | 3.42 | 3.21 |

**S24 Table Absolute expansion rate fitting results**

| Cement dosage  Coefficient | 5% | 7% | 9% |
| --- | --- | --- | --- |
| a | 0.0099 | 0.00863 | 0.00998 |
| b | -1.84133 | -1.59051 | -1.84646 |
| c | -0.02111 | -0.02112 | -0.02273 |
| d | 0.22394 | 0.23036 | 0.25191 |
| e | 85.56607 | 73.1972 | 85.28187 |
| R^2^ | 0.97363 | 0.97772 | 0.94861 |

**S25 Table Cement dose 5%. Fracture rate, absolute expansion, compaction fitting**

|  | Compaction/% | Fracture ratio/% | Absolute expansion/% |
| --- | --- | --- | --- |
| 1 dry wet cycle | 94 | 1.28 | 0.18 |
|  | 96 | 1.13 | 0.23 |
|  | 98 | 0.74 | 0.31 |
| 2 dry wet cycles | 94 | 2.41 | 0.38 |
|  | 96 | 2.05 | 0.39 |
|  | 98 | 1.46 | 0.46 |
| 3 dry wet cycles | 94 | 3.22 | 0.42 |
|  | 96 | 2.38 | 0.44 |
|  | 98 | 1.79 | 0.5 |
| 4 dry wet cycles | 94 | 3.93 | 0.47 |
|  | 96 | 3.44 | 0.51 |
|  | 98 | 2.32 | 0.52 |
| 5 dry wet cycles | 94 | 4.77 | 0.49 |
|  | 96 | 3.61 | 0.54 |
|  | 98 | 2.54 | 0.63 |
| 6 dry wet cycles | 94 | 5.12 | 0.54 |
|  | 96 | 3.99 | 0.55 |
|  | 98 | 2.85 | 0.65 |
| 7 dry wet cycles | 94 | 5.32 | 0.54 |
|  | 96 | 4.28 | 0.6 |
|  | 98 | 3.05 | 0.66 |

**S26 Table Cement dose 7%. Fracture rate, absolute expansion, compaction fitting**

|  | Compaction/% | Fracture ratio/% | Absolute expansion/% |
| --- | --- | --- | --- |
| 1 dry wet cycle | 94 | 1.41 | 0.24 |
|  | 96 | 1.25 | 0.27 |
|  | 98 | 0.87 | 0.36 |
| 2 dry wet cycles | 94 | 2.85 | 0.42 |
|  | 96 | 2.11 | 0.46 |
|  | 98 | 1.62 | 0.51 |
| 3 dry wet cycles | 94 | 3.33 | 0.46 |
|  | 96 | 2.89 | 0.51 |
|  | 98 | 2.05 | 0.63 |
| 4 dry wet cycles | 94 | 4.55 | 0.52 |
|  | 96 | 3.45 | 0.56 |
|  | 98 | 2.79 | 0.64 |
| 5 dry wet cycles | 94 | 4.76 | 0.54 |
|  | 96 | 3.98 | 0.62 |
|  | 98 | 2.96 | 0.7 |
| 6 dry wet cycles | 94 | 5.37 | 0.58 |
|  | 96 | 4.26 | 0.63 |
|  | 98 | 3.18 | 0.72 |
| 7 dry wet cycles | 94 | 5.45 | 0.57 |
|  | 96 | 4.52 | 0.65 |
|  | 98 | 3.42 | 0.76 |

**S27 Table Cement dose 9%. Fracture rate, absolute expansion, compaction fitting**

|  | Compaction/% | Fracture ratio/% | Absolute expansion/% |
| --- | --- | --- | --- |
| 1 dry wet cycle | 94 | 2.21 | 0.29 |
|  | 96 | 1.56 | 0.33 |
|  | 98 | 1.16 | 0.41 |
| 2 dry wet cycles | 94 | 2.96 | 0.47 |
|  | 96 | 2.24 | 0.49 |
|  | 98 | 2.21 | 0.58 |
| 3 dry wet cycles | 94 | 3.96 | 0.51 |
|  | 96 | 3.18 | 0.56 |
|  | 98 | 2.75 | 0.66 |
| 4 dry wet cycles | 94 | 4.57 | 0.53 |
|  | 96 | 4.25 | 0.62 |
|  | 98 | 3.33 | 0.71 |
| 5 dry wet cycles | 94 | 4.82 | 0.6 |
|  | 96 | 4.46 | 0.59 |
|  | 98 | 3.48 | 0.82 |
| 6 dry wet cycles | 94 | 5.57 | 0.58 |
|  | 96 | 4.85 | 0.65 |
|  | 98 | 3.7 | 0.79 |
| 7 dry wet cycles | 94 | 5.85 | 0.59 |
|  | 96 | 4.96 | 0.7 |
|  | 98 | 4.09 | 0.84 |

**S28 Table Absolute shrinkage rate fitting results**

| Cement dosage  Coefficient | 5% | 7% | 9% |
| --- | --- | --- | --- |
| f | 0.01143 | 0.00364 | -0.00156 |
| g | -2.12825 | -0.64979 | 0.35111 |
| h | -0.02876 | 0.00111 | -0.00987 |
| i | 0.26156 | 0.03985 | 0.16036 |
| j | 98.14199 | 28.38005 | -20.0691 |
| R^2^ | 0.95429 | 0.92301 | 0.97462 |

**S29 Table Cement dose 5%. Fracture rate, absolute shrinkage, compaction fitting**

|  | Compaction/% | Fracture ratio/% | Absolute shrinkage /% |
| --- | --- | --- | --- |
| 1 dry wet cycle | 94 | 1.28 | -0.63 |
|  | 96 | 1.13 | -0.59 |
|  | 98 | 0.74 | -0.55 |
| 2 dry wet cycles | 94 | 2.41 | -0.44 |
|  | 96 | 2.05 | -0.41 |
|  | 98 | 1.46 | -0.33 |
| 3 dry wet cycles | 94 | 3.22 | -0.41 |
|  | 96 | 2.38 | -0.37 |
|  | 98 | 1.79 | -0.28 |
| 4 dry wet cycles | 94 | 3.93 | -0.39 |
|  | 96 | 3.44 | -0.35 |
|  | 98 | 2.32 | -0.19 |
| 5 dry wet cycles | 94 | 4.77 | -0.38 |
|  | 96 | 3.61 | -0.28 |
|  | 98 | 2.54 | -0.18 |
| 6 dry wet cycles | 94 | 5.12 | -0.33 |
|  | 96 | 3.99 | -0.25 |
|  | 98 | 2.85 | -0.16 |
| 7 dry wet cycles | 94 | 5.32 | -0.32 |
|  | 96 | 4.28 | -0.24 |
|  | 98 | 3.05 | -0.15 |

**S30 Table Cement dose 7%. Fracture rate, absolute shrinkage, compaction fitting**

|  | Compaction/% | Fracture ratio/% | Absolute shrinkage /% |
| --- | --- | --- | --- |
| 1 dry wet cycle | 94 | 1.41 | -0.43 |
|  | 96 | 1.25 | -0.37 |
|  | 98 | 0.87 | -0.32 |
| 2 dry wet cycles | 94 | 2.85 | -0.43 |
|  | 96 | 2.11 | -0.37 |
|  | 98 | 1.62 | -0.32 |
| 3 dry wet cycles | 94 | 3.33 | -0.4 |
|  | 96 | 2.89 | -0.33 |
|  | 98 | 2.05 | -0.26 |
| 4 dry wet cycles | 94 | 4.55 | -0.35 |
|  | 96 | 3.45 | -0.3 |
|  | 98 | 2.79 | -0.21 |
| 5 dry wet cycles | 94 | 4.76 | -0.33 |
|  | 96 | 3.98 | -0.27 |
|  | 98 | 2.96 | -0.19 |
| 6 dry wet cycles | 94 | 5.37 | -0.28 |
|  | 96 | 4.26 | -0.26 |
|  | 98 | 3.18 | -0.17 |
| 7 dry wet cycles | 94 | 5.45 | -0.27 |
|  | 96 | 4.52 | -0.26 |
|  | 98 | 3.42 | -0.16 |

**S31 Table Cement dose 9%. Fracture rate, absolute shrinkage, compaction fitting**

|  | Compaction/% | Fracture ratio/% | Absolute shrinkage /% |
| --- | --- | --- | --- |
| 1 dry wet cycle | 94 | 2.21 | -0.54 |
|  | 96 | 1.56 | -0.49 |
|  | 98 | 1.16 | -0.44 |
| 2 dry wet cycles | 94 | 2.96 | -0.41 |
|  | 96 | 2.24 | -0.35 |
|  | 98 | 2.21 | -0.3 |
| 3 dry wet cycles | 94 | 3.96 | -0.35 |
|  | 96 | 3.18 | -0.27 |
|  | 98 | 2.75 | -0.25 |
| 4 dry wet cycles | 94 | 4.57 | -0.29 |
|  | 96 | 4.25 | -0.24 |
|  | 98 | 3.33 | -0.19 |
| 5 dry wet cycles | 94 | 4.82 | -0.26 |
|  | 96 | 4.46 | -0.21 |
|  | 98 | 3.48 | -0.15 |
| 6 dry wet cycles | 94 | 5.57 | -0.22 |
|  | 96 | 4.85 | -0.16 |
|  | 98 | 3.7 | -0.14 |
| 7 dry wet cycles | 94 | 5.85 | -0.21 |
|  | 96 | 4.96 | -0.15 |
|  | 98 | 4.09 | -0.12 |
